# Olfactory Enrichment of Captive Pygmy Hippopotamuses with Applied Machine Learning

**DOI:** 10.3390/ani16030385

**Published:** 2026-01-26

**Authors:** Jonas Nielsen, Frej Gammelgård, Silje Marquardsen Lund, Anja Sofie Banasik Præstekær, Astrid Vinterberg Frandsen, Camilla Strandqvist, Mikkel Haugaard Nielsen, Rasmus Nikolajgaard Olsen, Sussie Pagh, Thea Loumand Faddersbøll, Cino Pertoldi

**Affiliations:** 1 Department of Chemistry and Bioscience, Aalborg University, Frederik Bajers Vej 7H, 9220 Aalborg, Denmark; frga@aalborgzoo.dk (F.G.); sml@aalborgzoo.dk (S.M.L.); aprast23@student.aau.dk (A.S.B.P.); afrand23@student.aau.dk (A.V.F.); cstran23@student.aau.dk (C.S.); mhni23@student.aau.dk (M.H.N.); rolsen23@student.aau.dk (R.N.O.); sup@bio.aau.dk (S.P.); cp@bio.aau.dk (C.P.); 2 Aalborg Zoo, Mølleparkvej 63, 9000 Aalborg, Denmark; tlf@aalborgzoo.dk

**Keywords:** *Choeropsis liberiensis*, pose estimation, SLEAP, LabGym, animal welfare, olfactory enrichment, inter-observer variability, machine learning

## Abstract

The pygmy hippopotamus (*Choeropsis liberiensis*, Morton, 1849) is classified as Endangered by the International Union for the Conservation of Nature (IUCN). Compared to other large, threatened mammals, this species remains relatively understudied and new findings indicate potential welfare concerns, emphasizing the need for further research on the species welfare in zoological institutions. One approach to improving welfare in captivity is through environmental enrichment. This study investigated the effects of olfactory enrichment on three individual pygmy hippopotamuses through behavioral analysis and heat-map visualization. Using continuous focal sampling, several behaviors were influenced by the stimuli, with results showing a general decrease in inactivity and an increase in environmental engagement and interaction, particularly through scenting behavior. To further enhance behavioral quantification, machine learning techniques were applied to video data, comparing manual and automated behavior classification using the pose estimation program SLEAP. Four behaviors *Standing*, *Locomotion*, *Feeding/Foraging*, and *Lying Down* were compared. A confusion matrix, time budgets, and Kendall’s Coefficient of Concordance (W) were used to assess agreement between methods. The results showed a strong and moderate agreement between manual and automated annotations, for the female and calf, respectively. This demonstrates the potential of automation to complement behavioral observations in future welfare monitoring.

## Simple Summary

Zoologists and researchers commonly study animals by observing their behavior, enclosure use, and activity levels to gain insights into their engagement with the environment and their level of stimulation. Traditionally, this has been done by watching them directly, but such methods are time-consuming and often influenced by personal judgment. This study investigates the effects of scents as an enrichment on three individual pygmy hippopotamuses. Furthermore, we examined whether the software SLEAP (v1.4.1a2) could automate the tracking of the animals. Overall, the results indicated that the scents encouraged the hippos to explore more, and the software gave similar results to manual observations for most behaviors. This means that scent-based enrichment can be useful for pygmy hippopotamuses, and technology can help zoological institutions with monitoring animal welfare more easily.

## 1. Introduction

Captive management plays a key role in the survival of many threatened species, including the pygmy hippopotamus (*Choeropsis liberiensis*, Morton, 1849), which is currently listed as Endangered by the International Union for the Conservation of Nature (IUCN) [1,2]. While these populations help ensure survival, their welfare depends on appropriate husbandry and enrichment strategies [2,3]. Evidence-based approaches are essential to meet the species’ behavioral and sensory needs and to promote good welfare in zoological institutions [2,4,5].

For many years, it was generally assumed that the pygmy hippopotamus adapted well to captive conditions [6]. Early accounts from the beginning of the 20th century described the species as robust, manageable, and appeared to thrive under human care [6]. However, more recent observations have identified potential welfare-related concerns for individuals housed ex situ [3,6]. While this study does not constitute a formal welfare assessment, behavioral observations are widely used as welfare-relevant indicators in zoological research and can provide valuable insight into how animals respond to changes in husbandry and environmental enrichment [7,8,9].

One major concern for animals living in captivity is the potential lack of naturally occurring stimuli. Although many modern zoological institutions provide structurally complex and biologically enriched environments, certain sensory modalities, particularly olfactory stimulation, may still be underrepresented for some species, depending on enclosure design and husbandry practices [8,10]. For semi-aquatic species such as pygmy hippopotamuses, captive environments may be structurally adequate but still offer limited variation in sensory and exploratory stimuli, particularly with respect to olfactory enrichment, despite evidence of a well-developed olfactory system in hippopotamuses [10,11].

Olfactory enrichment is frequently applied as a form of sensory enrichment, as it targets the sense of smell, which plays a central role in foraging, mating, territorial marking, communication, and other ecologically relevant behaviors in many wildlife species [8,10,12]. In lowland tapirs (*Tapirus terrestris*, Linnaeus, 1758), olfactory enrichment has been suggested to be more effective than other enrichment modalities such as auditory or tactile enrichment [13]. Comparisons of ecological traits and habitat use between lowland tapirs and hippopotamuses suggest that olfactory cues may play an important role in environmental interaction in both taxa [8,13]. Furthermore, a study on common hippopotamuses (*Hippopotamus amphibius*, Linnaeus, 1758) reported that their olfactory system is highly developed and comparable to that of other artiodactyls, suggesting that olfactory-based enrichment could also be relevant for pygmy hippopotamuses [11]. To further examine the effects of olfactory enrichment in pygmy hippopotamuses, it is important to acknowledge individual variation (personality). This means that certain conditions or enrichment types may benefit one individual but not necessarily another [14,15]. In addition, behavioral patterns in this species have been shown to vary across the day, indicating that data collection should occur at different time points and over extended periods to provide a more accurate representation of individual behavior [16,17]. Research that incorporates these considerations has the potential to deepen our understanding of the species and improve the ability to provide appropriate environmental conditions and tailored enrichment strategies.

A commonly used method for studying behavior involves videographic recordings followed by manual annotation [18,19]. However, this approach is highly time-consuming, limits the ability to monitor multiple individuals simultaneously, and presents challenges for standardizing behavioral scoring across different observers (inter-observer variability) [20,21,22]. An emerging alternative is the application of machine learning to video data, in which a trained model can automatically detect behaviors and record them on a second-by-second basis. Such models estimate the animal’s pose by generating coordinate data from predefined anatomical points selected during training of the model [20,21,22,23]. Despite the promise of this approach, these methods remain in an early developmental phase, and further research is required to assess their reliability, accuracy, and practical usability in animal welfare studies.

This study investigates the behavior and interactions of three pygmy hippopotamuses and examines the effects of olfactory enrichment using essential oils. Behavioral patterns were assessed before and after enrichment, and a pose-estimation model for two individuals (mother and calf) was developed in SLEAP to compare automated scoring with manual scoring. This study examines the behavioral effects of enrichment and the agreement between manual and automated observations through the following hypotheses.

We predicted that olfactory enrichment would (1) lead to a redistribution of time budgets, characterized by a reduction in inactive behaviors and an increase in exploratory behavior and time spent on scent-directed behavior; (2) alter space use within the enclosure, with increased utilization of areas associated with scent placement. In addition, we predicted that (3) automated pose-estimation scoring would show a high degree of concordance with manual behavioral annotations, with discrepancies primarily arising from misclassification of subtle or transitional behaviors.

## 2. Materials and Methods

### 2.1. Subjects and Settings

The study was conducted on three pygmy hippopotamuses housed at Aalborg Zoo, consisting of one adult male (32 years), one adult female (13 years), and one calf (1 year). All individuals were born in captivity. The male originated from Lietuvos Zoologijos Sodas, Lithuania, and was transferred to Aalborg Zoo in 2000, while the female originated from Centre d’Etude et de Recherche Zoologique Augeron, France, and arrived at Aalborg Zoo in 2012. The calf was born at Aalborg Zoo in 2023. During the study period, the male was housed separately from the female and the calf.

**Figure 1:**
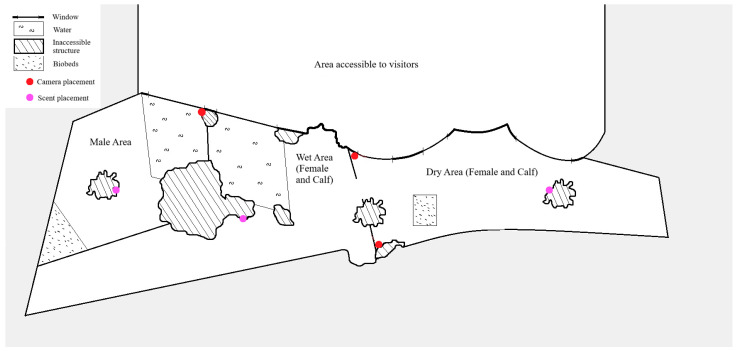
Schematic overview of the indoor enclosures at Aalborg Zoo, showing enclosure layout, camera placement, and scent placement (locations where olfactory stimuli were applied). The female and calf enclosure includes separate wet and dry areas, whereas the male enclosure consists of a single integrated space containing both wet and dry features.

All three individuals were fed a daily diet consisting of vegetables, hay, concentrated feed, browse sticks, and grass, with quantities adjusted according to age, body size, and seasonal requirements, following Aalborg Zoo husbandry protocols. The indoor enclosure measured approximately 46.5 m^2^ for the male and 139 m^2^ for the female and calf. The enclosure of the female and calf consisted of separate wet and dry areas, whereas the male enclosure comprised a single integrated area without distinct wet and dry zones. Due to decreasing outdoor temperatures, data collection focused exclusively on the indoor area, which was located within a tropical greenhouse maintained at 23 °C. A schematic overview of the enclosure is presented in Figure 1.

### 2.2. Data Collection

#### 2.2.1. Manual Observations

Behavioral observations for all three individuals were conducted across two sampling periods. The first period took place from the 9th to the 21st of October 2024 and served as a control period without added stimuli. The second period occurred from the 7th to the 15th of November 2024 and functioned as the test period in which olfactory enrichment was introduced. The enclosure was open to visitors from 10:00 to 17:00 in October and from 10:00 to 15:00 in November.

During the control period, behavior was recorded to establish baseline activity patterns. In the test period, olfactory enrichment was applied at three locations within the indoor enclosure: one in the male’s enclosure and two in the female and calf enclosure (one in the wet area and one in the dry area), see Figure 1. The individuals were exposed to four essential oils, *lemongrass*, *lavender*, *rosemary*, and *peppermint*, which were applied directly onto objects within the enclosure at the designated locations. Scents were applied one at a time for two days per scent placement, allowing voluntary investigation by the animals. Each scent remained in place until it was thoroughly removed the following day to avoid odor mixing, after which the next scent was introduced. The olfactory enrichment protocol, including the selection of essential oils, was reviewed and approved by Aalborg Zoo animal care staff prior to implementation.

#### 2.2.2. Camera Installation and Ethical Approval

Essential oils were applied only to objects within the enclosure and not directly to the animals, allowing interaction to occur without direct contact. The enrichment did not involve handling or changes to daily husbandry routines. The enrichment protocol and camera installation were implemented with Aalborg Zoo’s permission and following discussion with relevant zoo personnel (animal care staff and veterinary/research staff as appropriate) to ensure that procedures would not disturb the animals or interfere with normal husbandry. All cameras were installed prior to the observation periods and positioned outside the animals’ reach to avoid disturbance or interference with normal behavior.

All observations were recorded using three motion-activated trail cameras (two Bolyguard BG584 (Bolyguard, Shenzhen, China) and one Spromise S378 (Spomise, Shenzhen, China)). Two cameras monitored the enclosure of the female and the calf, while one monitored the male. When triggered by movement, each camera recorded 30 s video clips. Within each clip, behaviors were annotated on a per-second basis using continuous focal sampling, with the behavior performed in each second recorded according to the predefined ethogram (Appendix A). This approach allowed cumulative behavior durations to be quantified at a one-second temporal resolution. Camera placement is illustrated in Figure 1 and the most frequent blind spots are shown in Appendix B.

#### 2.2.3. Machine Learning and Processing

**Figure 2:**
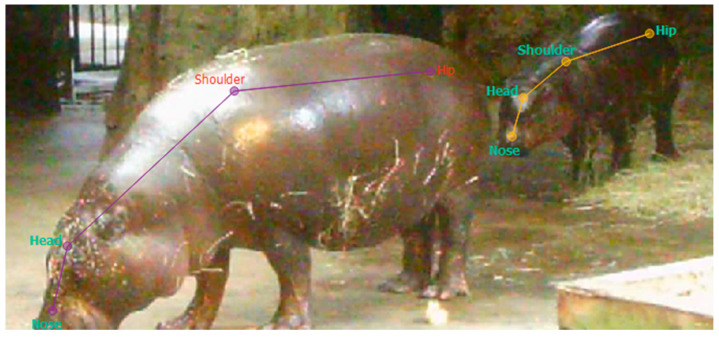
Example of a labeled frame from SLEAP with four body points nose, head, shoulder and hip.

Video footage was down-sampled in LabGym (v2.9.0) from 30 fps to 1 fps to reduce processing time and ensure comparability with manual observations at a one-second resolution. Training data were collected between 9th and 21st October 2024, and the final model was evaluated using 10 min of video footage recorded between 7th and 15th November 2024. Only footage from the dry enclosure housing the female and calf was used for comparison between manual observations and model predictions, as the pose-estimation model was trained and validated exclusively on this enclosure. Consequently, automated time budgets were generated only for the female and calf. The training dataset consisted of 127 labeled frames, which were annotated in SLEAP (Social LEAP Estimates Animal Poses, v1.4.1a2), a U-Net-based bottom-up neural network for pose estimation. Four body points were selected for tracking: *nose*, *head*, *shoulder*, *and hip*, see Figure 2.

The model was trained using a bottom-up approach, where body points for all individuals in the frame were detected simultaneously and subsequently grouped into separate animals. Training continued until validation performance plateaued, and the final model was selected based on the lowest validation loss. The bottom-up pose estimation model achieved its best validation loss of 0.000517 at epoch 38, where an epoch is one full pass through the training dataset and the validation loss represents the model’s error on unseen validation data. Tracking predictions were subsequently reviewed to ensure that tracks assigned to duplicates were removed. Once verified, the data were exported for behavioral analysis in RStudio (R version 4.3.2) [24].

#### 2.2.4. Comparison Between Manual and Automatic Observations

Body point coordinates for the nose, head, shoulder, and hip were used to classify behavior into four categories: *Standing*, *Lying Down*, *Foraging/Feeding*, and *Locomotion*. Examples of these categories are presented in Table 1. Behaviors were recorded on a per-second basis and incorporated into individual time budgets by summing the cumulative duration of each behavior and expressing it as a proportion of total observed time. The cumulative duration of each behavior for both the female and the calf was subsequently analyzed using Kendall’s Coefficient of Concordance (W) to assess agreement.

### 2.3. Analysis

#### 2.3.1. Manual Observations

Behavioral observations were analyzed and categorized using an ethogram (see Appendix A). Observations were conducted by six coders, and all data were recorded in Microsoft Excel (version 2410). Video footage was analyzed using continuous focal sampling [25,26]. Before data coding, an interobserver reliability test was performed in ZooMonitor (version 4.1) [27] to ensure agreement among the six coders. Interaction with olfactory enrichment was classified as part of the scenting behavior category.

For the male, the analyzed footage covered the period from the 9th to the 17th of October and the 7th to the 15th of November. For the female and calf, the footage covered the period from the 13th to the 21st of October and the 7th to the 15th of November. Specific values for the data collection can be seen in Appendix C.

#### 2.3.2. Model Performance

Before applying the SLEAP pose-estimation model to calculate time budgets, its reliability and performance first had to be evaluated. To do so, a confusion matrix was generated (Table 2), followed by an assessment based on five key performance metrics (Table 3): precision, accuracy, sensitivity, specificity, and true skill statistics (TSS).

The confusion matrix showed that *Standing*, *Lying Down*, and *Foraging/Feeding* were correctly predicted in most cases. The most frequent classification error for these behaviors was that they were not labeled, except for *Foraging/Feeding*, which was mostly misclassified as *Standing*. In contrast, the model performed poorly on *Locomotion*, which was predominantly misclassified as *Standing*. The category *Not Labeled* was correctly identified 286 times.

Precision was high for *Lying Down*, *Foraging/Feeding*, and *Locomotion*, indicating that when the model predicted one of these behaviors, it produced few false positives (Table 3). The opposite was the case with *Standing*. Accuracy was high across all behaviors, reflecting a large proportion of true values (true positives (TP) and true negatives (TN)) relative to all predictions. Sensitivity was high for *Lying Down*, but only moderate for *Standing*, *Foraging/Feeding*, and *Locomotion*. Specificity was high for all behaviors, showing that the number of TN was large relative to false positives (FP). Finally, TSS values were high for *Lying Down*, demonstrating strong predictive performance for this behavior, whereas the other three behaviors were near-random (0.5) or worse than random predictions.

### 2.4. Statistical Tests

#### 2.4.1. Manual Observations

Statistical analyses were conducted in RStudio (version 4.3.2) and Microsoft Excel (version 2410). Because repeated observations were collected from the same individuals across multiple days, analyses were conducted at the individual level, with control and test periods compared within each animal. Daily median values were used to reduce temporal autocorrelation and unequal sampling effort. Given the limited number of individuals, repeated-measures or mixed-effects models were not applied. As these data were not normally distributed, non-parametric tests were applied to account for skewness and potential outliers. χ^2^ tests were used where appropriate to assess differences in categorical behavioral distributions. Outliers were not removed because each behavioral observation was considered ecologically relevant and potentially influenced by environmental conditions. Data sets were constructed for each individual for both the control and test periods and contained the proportion of time spent on each behavior, expressed as a percentage of total observed time. Two data set formats were generated: (1) a single value per behavior for each period, and (2) daily values per behavior across each of the eight observation days within both periods. The second dataset provided increased statistical power by including daily medians. The behavioral category *Out of Sight* (or *Out of View*) was excluded from all analyses to ensure that only ecologically meaningful observations contributed to the results.

The proportion of time each individual engaged in each behavior was quantified first across the full control and test periods, and subsequently on a per-day basis. χ^2^ tests and Mann–Whitney *U*-tests were performed, as both are appropriate for analyzing non-parametric behavioral data while capturing different aspects of between-period differences. Specifically, Mann–Whitney *U*-tests were used to compare medians or ranked values of continuous measures, whereas χ^2^ tests were used to assess differences in categorical behavioral distributions. Together, these analyses provided a comprehensive evaluation of behavioral changes associated with olfactory enrichment. To assess whether behavioral variability differed between individuals and between control and test periods, a Fligner–Killeen test [28] was applied, comparing the dispersion of behavioral measures across groups. This non-parametric test evaluates homogeneity of variances and is robust to non-normal distributions.

#### 2.4.2. Machine Learning

The statistical analysis for the machine learning component was conducted using cumulative values derived from the time budgets, meaning that the total time spent on each behavior (e.g., *Standing*) in the manual observations was compared with the corresponding duration predicted by the automated model. A Kendall’s Coefficient of Concordance (W) test was used to compare the agreement between Manual and Model. The analysis was conducted in RStudio (version 4.3.2).

### 2.5. Heat Maps for Manual Observations

Heat maps were generated using RStudio (version 4.3.2) and Fiji (version 2.16.0) [29]. A digital blueprint of the enclosure enabled spatial tracking of each individual. Within each video clip, a tracking point was recorded in Fiji every 15 s when the individual was visible. Using the ROI Manager tool in Fiji, each tracking point was converted into an XY-coordinate on the enclosure blueprint. These coordinates were subsequently imported into RStudio, where the heat maps were produced. Heat maps were generated exclusively from manual observations and not from machine learning based tracking, as the automated pose-estimation analysis was limited to terrestrial behaviors. The resulting visualizations illustrate individual movement patterns by displaying areas of higher and lower spatial use based on the density of recorded tracking points. Heat maps are visualized in Section 3.1.4 Heat Maps and Appendix D.

## 3. Results

### 3.1. Manual Observations

#### 3.1.1. Proportion of Time Spent on Each Behavior

**Figure 3:**
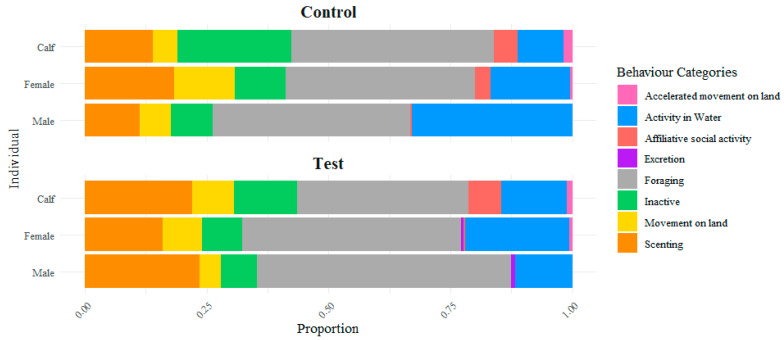
Proportion of time spent on each behavior during the control (**top**) and test (**bottom**) periods. Each period contains three bars representing the three individuals, with each bar showing the relative proportion of time allocated to each behavior.

Time spent on each behavior varies between individuals and across observational periods. Figure 3 provides a summary of the proportional distribution of behavioral categories for all individuals within each period. Overall, the calf exhibited a higher proportion of inactive behaviors compared to the other individuals. In contrast, the male showed a relatively large proportion of time allocated to water-related activity during the control period, exceeding both the female and the calf. However, during the test period, the male displayed a reduction in time spent on water activity, resulting in the lowest proportional allocation among the three individuals. These differences are examined in greater detail in the subsequent section.

#### 3.1.2. χ^2^-Test and Mann–Whitney U-Test

Differences in the proportion of time allocated to each behavior, both between individuals and between control and test periods, were evident across all behavioral categories except *Foraging*, for which no significant differences were detected, see Appendix E and Appendix F. During the control period, the male spent significantly more time engaged in water-related activity compared to both the female (*p* < 0.05) and the calf (*p* < 0.01) (Appendix E.2). A significant difference in time spent on water-related activity was also found between periods, with the male exhibiting a decrease (*p* < 0.01) (Appendix E.1) and the female an increase (*p* < 0.05) (Appendix F.1), during the test period. During the control period, both the male (*p* < 0.01) and the female (*p* < 0.05) spent significantly less time in inactive behaviors compared to the calf (Appendix E.2). Following exposure to olfactory enrichment, all individuals demonstrated an increase in *Scenting* behavior (Appendix G), with this change reaching significance for the male (*p* < 0.01) (Appendix F.1).

#### 3.1.3. Fligner-Killeen Test

Only a limited number of significant differences were detected using the Fligner–Killeen test (Appendix H) when comparing the control and test periods for each individual. For the male, a significant decrease in the interquartile range (IQR) for *Activity in Water* was observed in the control period compared to the test period (*p* < 0.05) (Appendix H.1). In contrast, multiple significant differences were identified when comparing individuals during the control period. The male exhibited a significantly higher IQR for *Activity in Water* compared to both the female and the calf (*p* < 0.01) (Appendix H.2). Additionally, the calf displayed a significantly higher IQR for *Inactive* behavior relative to both the male and the female (*p* < 0.05) (Appendix H.2). However, during the test period, almost no significant differences were detected between individuals (Appendix H.3).

#### 3.1.4. Heat Maps

**Figure 4:**
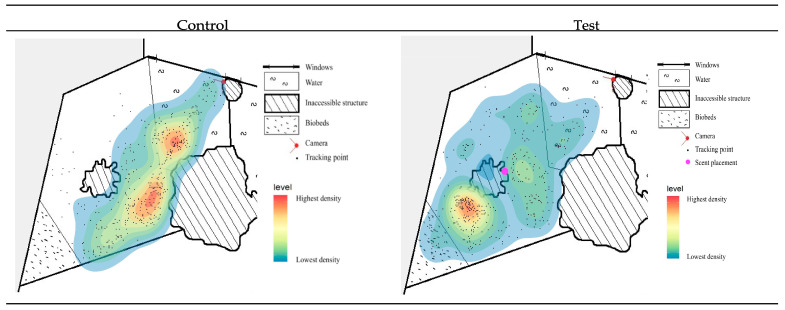
Heat maps illustrating the spatial distribution and movement frequency of the male pygmy hippopotamus. The maps display the density of recorded tracking points. Red indicates the highest density and blue indicates the lowest density.

**Figure 5:**
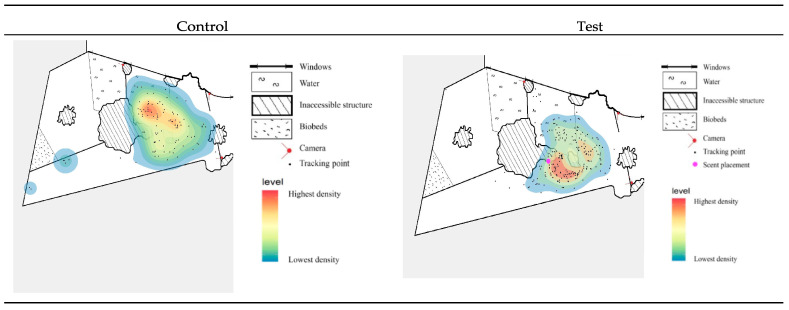
Heat maps illustrating the spatial distribution and movement frequency of the pygmy hippopotamus calf within the wet area. Red indicates the highest density, and blue indicates the lowest density of tracking points.

To assess potential changes in enclosure utilization between the control and test periods, heat maps were constructed for each individual (Figure 4 and Figure 5 and Appendix D). For the male, the heat map demonstrated a higher density of recorded locations along the water’s edge and within the water during the control period compared to the test period. In contrast, the test period revealed an increased density of locations in front of the biobed, and overall movement appeared more evenly distributed relative to the control period (see Figure 4).

For the female, no substantial differences in enclosure utilization were observed when comparing the control and test periods. The density of tracking points in the dry area appeared more dispersed during the control period compared to the test period (Appendix D.1). Nevertheless, the areas of highest density within both the dry and wet zones remained approximately unchanged across periods (Appendix D.1 and Appendix D.2).

In contrast, the heat map representing the calf’s movement indicated minor spatial changes between the two periods. During the test period, a higher density of tracking points was recorded across a broader area in front of the biobed compared to the control period, see Figure 5. Additionally, within the wet area, the location of the highest density shifted towards the scent-enriched site during the test period.

### 3.2. Machine Learning

#### 3.2.1. Activity Tracking for the Female

To compare manual observations with the model-generated predictions, separate time budgets were produced for the female and the calf.

**Figure 6:**
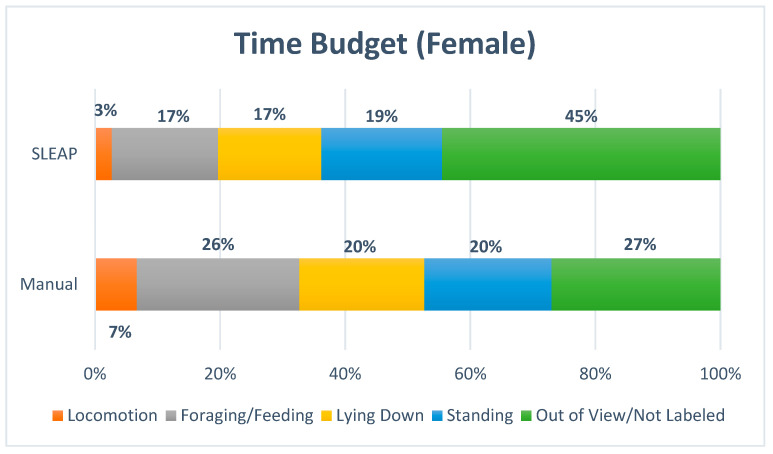
The time budget for the female displayed the percentage of time allocated to each of the four behaviors, Locomotion, Foraging/Feeding, Lying Down, Standing and with Out of View/Not Labeled included.

**Figure 7:**
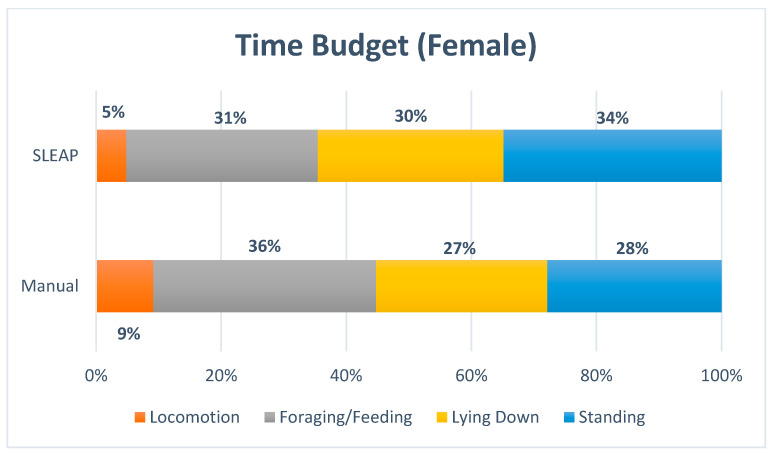
The time budget for the female displayed the percentage of time allocated to each of the four behaviors: Locomotion, Foraging/Feeding, Lying Down and Standing. Out of View/Not Labeled was excluded.

The time budget (Figure 6 and Figure 7) indicated a strong similarity between manual observations and automated predictions for *Lying Down*, whereas lower agreement was observed for *Standing, Locomotion* and *Foraging/Feeding*. The proportion of frames classified as *Out of View/Not Labeled* was nearly doubled by SLEAP, likely reflecting missed annotations, particularly for *Foraging/Feeding* and *Locomotion*.

The proportion of time allocated to each behavior was evaluated using Kendall’s Coefficient of Concordance (W), which resulted in a W value of 0.95 and a *p*-value of 0.107. This suggests a very high level of agreement between manual and model-derived classifications; however, the result did not reach statistical significance, likely due to the limited number of behavioral categories included in the analysis.

#### 3.2.2. Activity Tracking for the Calf

**Figure 8:**
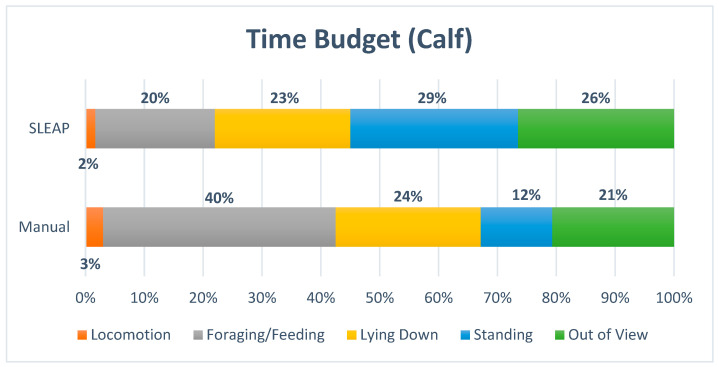
The time budget for the calf displayed the percentage of time allocated to each of the four behaviors, Locomotion, Foraging/Feeding, Lying Down, Standing and with Out of View/Not Labeled included.

**Figure 9:**
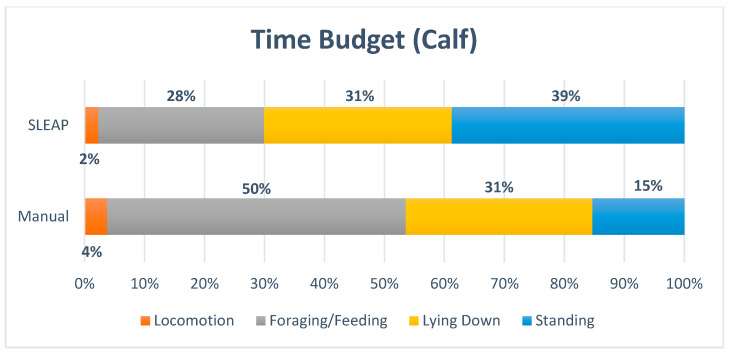
The time budgets, for the calf displayed the percentage of time allocated to each of the four behaviors: Locomotion, Foraging/Feeding, Lying Down and Standing. Out of View/Not Labeled was excluded.

The time budget (Figure 8 and Figure 9) indicated strong similarity between manual observations and automated predictions for *Lying Down*. In contrast, lower agreement was observed for *Locomotion*, *Standing*, and *Foraging/Feeding*. SLEAP reduced the proportion of frames classified as *Foraging/Feeding* by approximately half, which likely reflects misclassification, primarily into the *Standing* category.

The proportion of time allocated to each behavior was evaluated using Kendall’s Coefficient of Concordance (W), resulting in a W value of 0.50 and a *p*-value of 0.406. This suggests only low to moderate agreement between manual and model-derived classifications, and the result was not statistically significant.

## 4. Discussion

### 4.1. Manual Observations

#### 4.1.1. Behavioral Responses to Olfactory Enrichment

The results of this study demonstrated that olfactory enrichment influenced behavioral patterns in all three pygmy hippopotamuses housed at Aalborg Zoo, as behavioral differences were observed between the control and test periods. Significant increases in activity-related behaviors were found, particularly for the male. For this individual, the reduction in time spent in water, which previously was the primary location for passive behavior, indicates a shift towards a more active behavioral profile following exposure to the olfactory stimuli. Additionally, *Scenting* behavior increased significantly for the male during the test period, suggesting heightened environmental engagement. An increase in *Scenting* behavior was also observed for the female and calf, indicating a generalized response to the enrichment (Appendix G). As changes in one behavior necessarily affect the allocation of time to others, it is notable that the calf showed a significant reduction in inactive behavior. Given that the calf spent significantly more time in inactive behaviors than the adults during the control period, this reduction may be interpreted as a potentially beneficial shift towards a more active state. However, the high level of inactivity in the control period may be age-related, as younger individuals often require longer resting periods than adults [30].

Despite this general trend, the three individuals displayed differing behavioral responses to the enrichment. Results from the Fligner–Killeen test revealed significant inter-individual variability in the dispersion of behavioral measures (Appendix H). This variation highlights that even conspecifics, housed under identical conditions and exposed to identical stimuli, may respond differently to environmental changes. Such findings support the importance of considering individual differences in welfare monitoring and the potential need for tailored individual-focused management strategies within zoological settings.

The heat maps further illustrate changes in enclosure use following enrichment, providing spatial context to the behavioral observations. The male showed increased use of terrestrial areas during the test period (Figure 4), consistent with the reduction in water-based behavior. This terrestrial shift aligns more closely with natural activity patterns documented for pygmy hippopotamuses in the wild [31], which may tentatively suggest that the enrichment promoted more species-typical space use. For the calf, movement patterns also appeared to be influenced by the enrichment, particularly within the wet area of the enclosure, where movement density increased around the location of the olfactory stimulus (Figure 5). This suggests a clear spatially directed response to the enrichment.

In contrast, minimal differences were observed for the female, as heat maps showed comparable space-use patterns across periods (Appendix D.1 and Appendix D.2). This lack of response further reinforces the presence of individual variation. Additionally, the female contributed the fewest tracking points and had the shortest observation time, which may have influenced the ability to detect significant changes.

#### 4.1.2. Reliability of the Olfactory Enrichment

The three individuals in this study were housed in two enclosures of differing sizes, with the female and calf having substantially more space available than the male (Figure 1). This discrepancy reduced the likelihood of observing certain behaviors in the male, such as rapid terrestrial movement and affiliative social interactions, and therefore limited the direct comparability of these behaviors between individuals. Furthermore, the findings cannot be generalized to all captive pygmy hippopotamuses, as this case study reports on only three individuals at Aalborg Zoo.

The study was conducted across two observation periods, resulting in a total duration of 16 days and 97.960 s of video footage for all individuals combined. As the cameras were motion-activated, variation occurred in the total recorded observation time across individuals (Appendix C).

Research on pygmy hippopotamuses remains scarce, particularly regarding olfactory enrichment [3,6]. Consequently, this study represents the first and, to date, most extensive investigation into the behavioral effects of olfactory enrichment in this species. Despite the relatively short duration, the study yielded several significant results, and the datasets produced provided an important initial reference point for future research. Thus, the findings presented here should be regarded as preliminary, contributing foundational knowledge to the emerging field of scent-based enrichment for pygmy hippopotamuses.

The results indicate that olfactory enrichment may influence behavior in captive pygmy hippopotamuses. While this is an important finding, several considerations should be addressed in future studies. One key direction for further research concerns the type and specificity of olfactory stimuli. Previous work has shown that different essential oils can elicit distinct behavioral responses, suggesting that targeted evaluation of individual scent types may allow for refinement and optimization of enrichment protocols [8].

Another important consideration is the potential for habituation, which may emerge over time but could be mitigated by implementing variation in enrichment type and delivery [32]. Investigating such patterns in pygmy hippopotamuses would provide valuable insight into the long-term effectiveness and sustainability of olfactory enrichment practices.

Future research could also be strengthened by increasing the length of the study period, expanding data collection across multiple seasons, and increasing sample size. Taken together, the results from this case study, combined with considerations regarding stimulus type, habituation, and study design improvements, suggest that olfactory enrichment holds promise as a potentially beneficial addition to sensory-based husbandry practices for pygmy hippopotamuses.

### 4.2. Results of Applied Pose Estimation

The machine learning results indicate strong potential for certain behavioral categories, while performance remains limited for others. Based on the confusion matrix, *Lying Down* appears to be highly detectable, reflecting good model performance for this behavior. This is supported by the calculated performance metrics, particularly the TTS value of 0.827, which demonstrates strong predictive capability. The findings were further reflected not only in the confusion matrix but also in the similarity between manual and automated time budgets, indicating a high level of correspondence between the two methods. In contrast, *Locomotion* and *Foraging/Feeding* showed weaker performance, with frequent misclassification as *Standing* and a substantial increase in *Not Labeled* frames. Notably, discrepancies between manual and SLEAP time budgets were most pronounced for Foraging/Feeding, likely because subtle head- and mouth-directed movements can be difficult to distinguish from stationary standing postures using pose-based features alone. This is also reflected in the time budgets for the two individuals. A key limitation was the substantial proportion of Out of View/Not Labeled footage, suggesting that future work may benefit from refined camera placement and sampling design to reduce occlusion and improve behavioral inference across species.

Agreement analyses further supported these findings. For one individual, Kendall’s W indicated very high but non-significant agreement between manual and automated scoring, while for the other, it suggested only low to moderate concordance. This difference highlights that model performance may vary not only by behavior type but also by dataset characteristics such as visibility, pose diversity, and individual movement style.

Collectively, the findings indicate that pose estimation can serve as a valuable complement to manual scoring for easily distinguishable behaviors and may, under certain conditions, have the potential to replace manual observations. Expanding the training dataset, including additional key body parts, and testing across varied conditions may improve performance. Comparable results have been reported in studies employing pose estimation and object detection, where automated methods demonstrate clear strengths for some behavior types but show limited performance for certain behavior types [20,21,22].

## 5. Conclusions

This study provides preliminary evidence that olfactory enrichment can influence behavior and space use in captive pygmy hippopotamuses, with increases in activity-related and scent-directed behaviors observed during the enrichment period. The enrichment appeared particularly effective for the male, who shifted from predominantly water-based inactivity to more terrestrial exploration and increased scent-directed interaction, suggesting enhanced behavioral diversity and potentially more species-typical use of space. However, responses differed between individuals, underscoring the importance of personalized welfare assessment and the need to consider factors such as age, enclosure layout, and observation effort when interpreting enrichment effects.

The application of pose estimation showed promising performance for clearly distinguishable behaviors but was less reliable for subtle or movement-based behaviors, indicating behavior-specific model sensitivity. With improvements such as larger and more diverse training datasets, additional anatomical key points, and testing under broader conditions, automated behavioral analysis could serve as a complementary tool and, for certain behaviors, potentially replace manual observation. A notable limitation was the substantial proportion of out-of-view footage, indicating that refined camera placement and sampling may be needed in future studies.

Overall, the study contributes novel baseline knowledge on both olfactory enrichment and automated behavioral assessment in pygmy hippopotamuses, highlighting encouraging potential, methodological considerations, and the value of further multi-institutional and long-term research.

## Figures and Tables

**Table 1:** Classification criteria for terrestrial behaviors based on positional and movement data across video frames. Only terrestrial behaviors were included in the automated classification for the female and calf.

Behavior	Condition
Foraging/Feeding	If head position is at least 60 pixels below the shoulder, or if nose is at least 90 pixels below shoulder.
Locomotion	Displacement of hip and shoulder exceeded one half of a body length (hip-to-shoulder distance) between two frames. The behavior must be initially identified as Standing.
Lying Down	If the hip or shoulder coordinates were located within the following boundaries x_min = 1080, x_max = 1720, y_min = 270 and y_max = 400.
Standing	If no other behavior is qualified (default behavior).

**Table 2:** A multi-class confusion matrix showing a comparison between manual coding and model prediction for each behavior. A more intense color represents a stronger correlation.

	Manually Observed					
Predicted by SLEAP	**Behavior:**	**Standing**	**Lying Down**	**Foraging/Feeding**	**Locomotion**	**Out of View**
	Standing	100	2	158	27	
	Lying Down	9	225	3		
	Foraging/Feeding	1		221	2	
	Locomotion				26	
	Not labeled	85	41	11	3	286
	Sum:	195	268	393	58	286

**Table 3:** Key metrics of the multi-class confusion matrix, showing model performance for each behavior. Summary of overall model performance metrics across female and calf.

Metrics	Standing	Lying Down	Foraging/Feeding	Locomotion	Out of View
Precision	0.348	0.949	0.987	1	0.671
Accuracy	0.765	0.954	0.854	0.973	0.883
Sensitivity	0.513	0.840	0.562	0.448	1
Specificity	0.814	0.987	0.996	1	0.847
TSS	0.327	0.827	0.559	0.448	0.847

**Figure A1:**
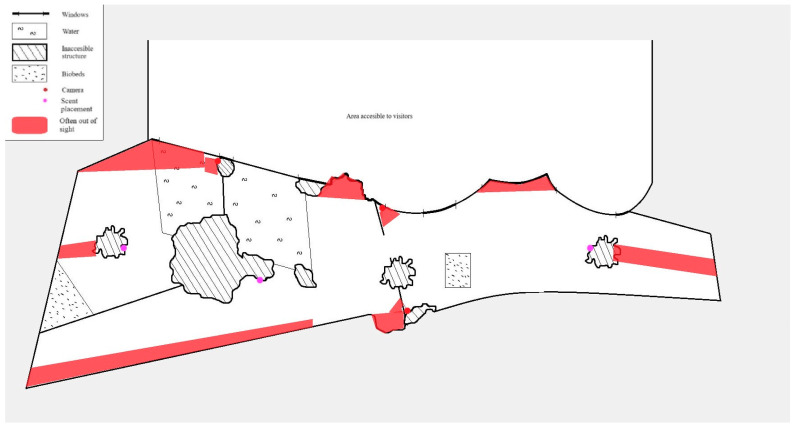
The map of the pygmy hippopotamuses indoor enclosure at Aalborg Zoo, with the blind spots marked in red.

**Figure A2:**
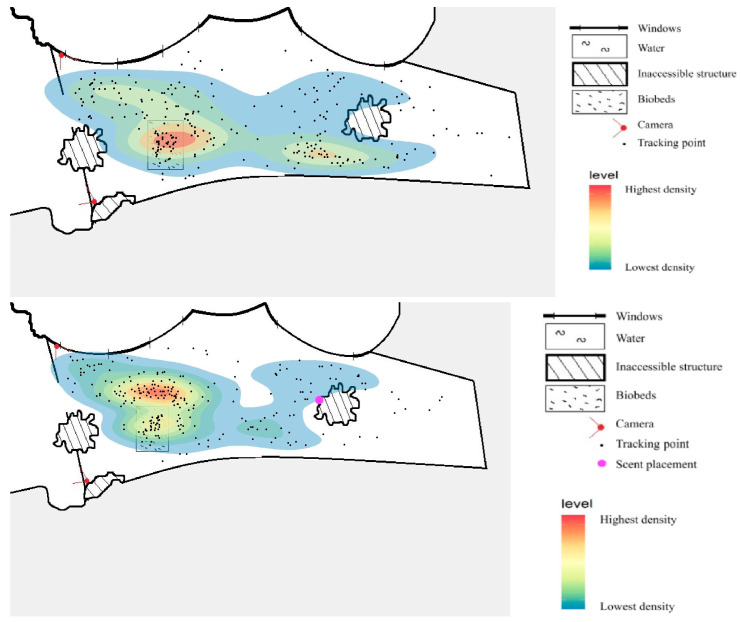
The heat maps show the female’s movement patterns in the dry area during the control period (**top**) and the test period (**bottom**). The map illustrates the density of recorded tracking points, with red indicating the highest density and blue the lowest.

**Figure A3:**
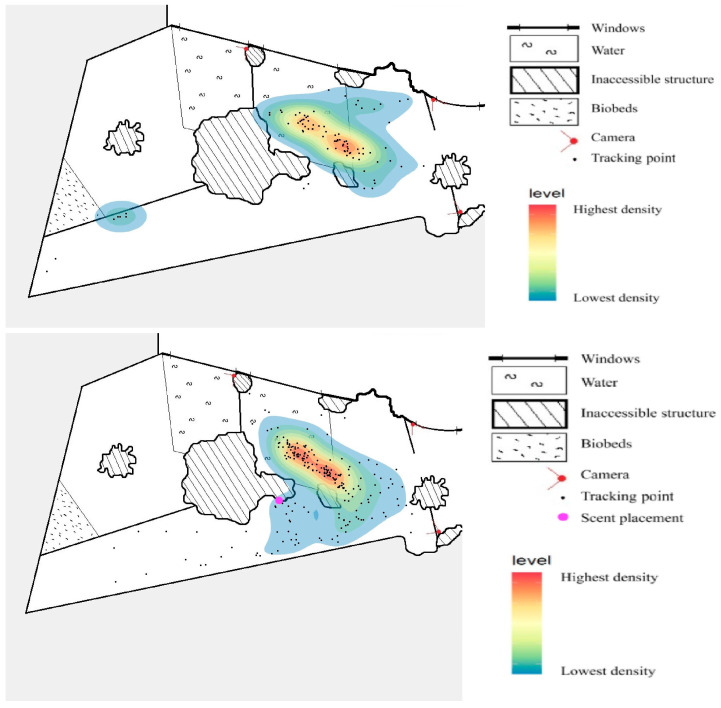
The heat maps show the female’s movement patterns in the wet area during the control period (**top**) and the test period (**bottom**). The map illustrates the density of recorded tracking points, with red indicating the highest density and blue the lowest.

**Figure A4:**
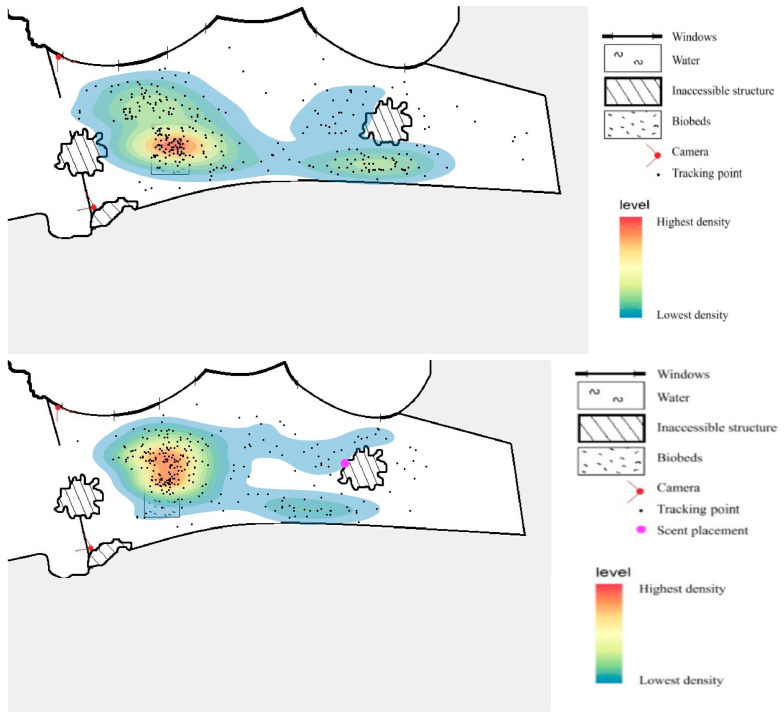
The heat maps show the calf’s movement patterns during the control period (**top**) and the test period (**bottom**). The map illustrates the density of recorded tracking points, with red indicating the highest density and blue the lowest.

**Figure A5:**
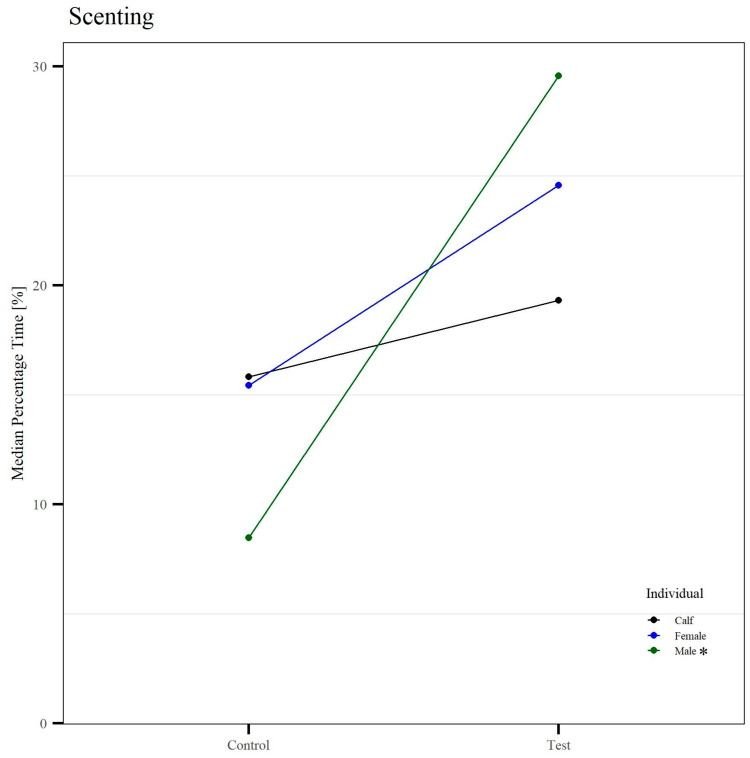
For each individual, the median percentage of time spent on scenting was calculated for the control and test periods. A trend line connects the two medians to illustrate whether scenting increased or decreased across periods. A significant difference between the control and test period for a given individual is indicated by an asterisk (*).

## Data Availability

The data presented in this study are available on request from the corresponding author.

## Appendix A

### Behavioral Ethogram

**Table A1:** Ethogram Used for Behavioral Coding of the Three Subjects.

Behavioral Category	Active Behavior	Definition
Inactive	Standing Resting on land	All four legs on the ground with minimal movement, and the head positioned above the front legs. Being on either belly, or on the side, without using legs as the primary support.
Movement on Land	Scenting Scratching Walking	The movement in which the snout is held close to the ground without eating or drinking, accompanied by active head motion. The motion of rubbing the body against a rough or textured surface. The movement in which only one limb is lifted from the ground at a time.
Accelerated Movement on Land	Running Jumping	A gait in which at least two limbs, one anterior and one posterior, are lifted from the ground simultaneously. The movement in which both anterior and/or posterior limbs are lifted from the ground simultaneously.
Foraging	Eating Drinking	Ingestion or chewing on food. Ingestion of water.
Affiliative Social Activity	Social Interaction Following Chasing	Two individuals engage in social interaction, either through playful behavior or physical contact. One individual moving directly behind another while walking, following the same path or movement pattern. One individual moving directly behind another while running, following the same path or movement pattern.
Agonistic Social Activity	Aggressive Behavior	One individual harming or attempting to harm another, such as through biting or pushing.
Activity in Water	Activity in Water	All behaviors exhibited in water, including swimming, diving, standing, resting, and related activities.
Excretion	Excretion	Urination or defecation
Out of Sight	Out of Sight	Out of sight due to blind spots.
Other	Other	Any behaviors not otherwise defined within the ethogram.

## Appendix C

### Collected Data

**Table A2:** An overview of the collected data for the male, female, and calf. Three types of data are presented: total observation time, number of tracking points, and number of recorded behavioral events. The bottom row shows the summed values.

Overview of Data	Time (s)	Tracking Points	Amount of Behavior
Male	41.360	943	2278
Female	26.160	806	1989
Calf	30.440	925	1292
Sum	97.960	2674	5559

## Appendix E

### Appendix E.1. χ^2^-Test Comparing Control vs. Test

**Table A3:** χ^2^-test results examining significant differences in the median time allocated to each behavior when comparing the control period with the test period. Cells marked with NA indicate behaviors observed less than 5% and NS indicate not significant.

Behavior	Male	Female	Calf
Activity in Water	χ^2^ = 10.04 *p* < 0.01	χ^2^ = 0.7472 NS	χ^2^ = 0.7157 NS
Foraging	χ^2^ = 1.470 NS	χ^2^ = 0.4281 NS	χ^2^ = 0.5455 NS
Inactive	χ^2^ = 0.0945 NS	χ^2^ = 0.2781 NS	χ^2^ = 2.932 NS
Movement on Land	NA	χ^2^ = 0.9222 NS	χ^2^ = 0.8736 NS
Scenting	χ^2^ = 4.427 NS	χ^2^ = 0.1490 NS	χ^2^ = 1.827 NS

### Appendix E.2. χ^2^-Test Comparing Individuals in Control Period

**Table A4:** χ^2^-test results examining significant differences in the median time allocated to each behavior when comparing individuals in the control period. Cells marked with NS indicate not significant.

Behavior	Male vs. Female	Male vs. Calf	Female vs. Calf
Activity in Water	χ^2^ = 5.641 *p* < 0.05	χ^2^ = 13.03 *p* < 0.001	χ^2^ = 1.820 NS
Foraging	χ^2^ = 0.0305 NS	χ^2^ = 0.0131 NS	χ^2^ = 0.0837 NS
Inactive	χ^2^ = 0.1803 NS	χ^2^ = 6.847 *p* < 0.01	χ^2^ = 0.4953 *p* < 0.05
Movement on Land	χ^2^ = 1.876 NS	χ^2^ = 0.1736 NS	χ^2^ = 3.106 NS
Scenting	χ^2^ = 1.701 NS	χ^2^ = 0.3047 NS	χ^2^ = 0.5769 NS

### Appendix E.3. χ^2^-Test Comparing Individuals in Test Period

**Table A5:** χ^2^-test results examining significant differences in the median time allocated to each behavior when comparing individuals in the test period. Cells marked with NA indicate behaviors observed less than 5% and NS indicate not significant.

Behavior	Male vs. Female	Male vs. Calf	Female vs. Calf
Activity in water	χ^2^ = 2.862 NS	χ^2^ = 0.1178 NS	χ^2^ = 0.1844 NS
Foraging	χ^2^ = 0.5396 NS	χ^2^ = 3.351 NS	χ^2^ = 1.215 NS
Inactive	χ^2^ = 0.0411 NS	χ^2^ = 1.581 NS	χ^2^ = 1.124 NS
Movement on land	NA	NA	χ^2^ = 0.0107 NS
Scenting	χ^2^ = 1.458 NS	χ^2^ = 0.5113 NS	χ^2^ = 0.9651 NS

## Appendix F

### Appendix F.1. Mann-Whitney U-Test Comparing Control vs. Test

**Table A6:** Mann–Whitney U-test results examining significant differences in the median time allocated to each behavior when comparing control period with test period. Cells marked with NA indicate behaviors observed less than 5%, and NS indicates not significant.

Behavior	Male	Female	Calf
Accelerated Movement on Land	NA	W = 35.0 NS	W = 32.0 NS
Activity in Water	W = 50.0 NS	W = 11.0 *p* < 0.05	W = 20.0 NS
Affiliative Social Activity	W = 36.0 NS	W = 48.0 NS	W = 13.0 NS
Agonistic Social Activity	NA	NA	NA
Excretion	W = 12.0 *p* < 0.05	W = 23.5 NS	NA
Foraging	W = 29.0 NS	W = 46.0 NS	W = 40.0 NS
Inactive	W = 30.0 NS	W = 41.0 NS	W = 46.0 NS
Movement on land	W = 27.0 NS	W = 38.0 NS	W = 17.0 NS
Scenting	W = 6.0 *p* < 0.01	W = 26.0 NS	W = 20.0 NS

### Appendix F.2. Mann–Whitney U-Test Comparing Individuals in Control Period

**Table A7:** Mann–Whitney U-test results examining significant differences in the median time allocated to each behavior when comparing individuals in control period. Cells marked with NA indicate behaviors observed less than 5%, and NS indicates not significant.

Behavior	Male vs. Female	Male vs. Calf	Female vs. Calf
Accelerated Movement on Land	W = 16.0 *p* < 0.05	W = 8.0 *p* < 0.01	W = 19.0 NS
Activity in Water	W = 49.0 NS	W = 55.0 *p* < 0.05	W = 39.0 NS
Affiliative Social Activity	W = 14.5 *p* < 0.05	W = 2.0 *p* < 0.01	W = 23.0 NS
Agonistic Social Activity	NA	NA	NA
Excretion	W = 28.0 NS	NA	W = 36.0 NS
Foraging	W = 29.0 NS	W = 27.0 NS	W = 29.0 NS
Inactive	W = 15.0 NS	W = 5.0 *p* < 0.01	W = 12.0 *p* < 0.05
Movement on Land	W = 13.0 NS	W = 24.0 NS	W = 53.0 *p* < 0.05
Scenting	W = 13.0 NS	W = 20.0 NS	W = 40.0 NS

### Appendix F.3. Mann–Whitney U-Test Comparing Individuals in Test Period

**Table A8:** Mann–Whitney U-test results examining significant differences in the median time allocated to each behavior when comparing individuals in test period. Cells marked with NA indicate behaviors observed less than 5%, and NS indicates not significant.

Behavior	Male vs. Female	Male vs. Calf	Female vs. Calf
Accelerated Movement on Land	W = 20.0 NS	W = 4.0 *p* < 0.01	W = 17.5 *p* = 0.1311
Activity in Water	W = 13.0 NS	W = 28.0 NS	W = 44.0 *p* = 0.2271
Affiliative Social Activity	W = 24.0 NS	W = 0 *p* < 0.001	W = 0 *p* < 0.001
Agonistic Social Activity	NA	NA	NA
Excretion	W = 38.5 NS	W = 52.0 *p* < 0.05	W = 44.0 NS
Foraging	W = 46.0 NS	W = 15.0 NS	W = 18.0 NS
Inactive	W = 28.0 NS	W = 15.0 NS	W = 18.0 NS
Movement on Land	W = 15.0 NS	W = 14.0 NS	W = 29.0 NS
Scenting	W = 42.0 NS	W = 43.0 NS	W = 40.0 NS

## Appendix H

### Appendix H.1. Fligner-Killeen Test Comparing Control vs. Test

**Table A9:** Fligner-Killeen test results assessing significant difference in variability for each behavior when comparing control period with test period. Cells marked with NA indicate behaviors observed less than 5%, and NS indicates not significant.

Behavior	Male	Female	Calf
Accelerated movement on Land	NA	T = 10.89 NS	T = 7.014 NS
Activity in Water	T = 9.369 *p* < 0.05	T = 0.0126 NS	T = 2.258 NS
Affiliative Social Activity	T = 1.000 NS	T = 9.322 *p* < 0.01	T = 0.2686 NS
Excretion	T = 10.98 *p* < 0.001	T = 1.505 NS	NA
Foraging	T = 0.0420 NS	T = 0.8191 NS	T = 0.6933 NS
Inactive	T = 0.0530 NS	T = 0.5325 NS	T = 6.153 *p* < 0.05
Movement on Land	T = 0.7827 NS	T = 0.4009 NS	T = 0.5518 NS
Scenting	T = 0.2682 NS	T = 2.564 × 10^−3^ NS	T = 0.0485 NS

### Appendix H.2. Fligner-Killeen Test Comparing Individuals in Control Period

**Table A10:** Fligner-Killeen test results assessing significant difference in variability for each behavior when comparing individuals in control period. Cells marked with NA indicate behaviors observed less than 5%, and NS indicates not significant.

Behavior	Male vs. Female	Male vs. Calf	Female vs. Calf
Accelerated Movement on Land	T = 11.11 *p* < 0.001	T = 1.547 *p* < 0.01	T = 1.547 *p* < 0.05
Activity in Water	T = 7.177 *p* < 0.01	T = 9.046 *p* < 0.01	T = 1.282 NS
Affiliative Social Activity	T = 7.427 *p* < 0.001	T = 8.144 *p* < 0.001	T = 0.0141 NS
Excretion	T = 1.000 NS	NA	T = 1.000 NS
Foraging	T = 1.393 NS	T = 2.884 NS	T = 0.3992 NS
Inactive	T = 0.0114 NS	T = 3.979 *p* < 0.05	T = 5.012 *p* < 0.05
Movement on Land	T = 0.4212 NS	T = 0.5516 NS	T = 0.0806 NS
Scenting	T = 0.3711 NS	T = 5.468 × 10^−4^ NS	T = 1.064 NS

### Appendix H.3. Fligner-Killeen Test Comparing Individuals in Test Period

**Table A11:** Fligner-Killeen test results assessing significant difference in variability for each behavior when comparing individuals in test period. Cells marked with NS indicates not significant.

Behavior	Male vs. Female	Male vs. Calf	Female vs. Calf
Accelerated Movement on Land	T = 3.286 NS	T = 10.87 *p* < 0.001	T = 1.547 NS
Activity in Water	T = 0.0116 NS	T = 0.8528 NS	T = 0.5899 NS
Affiliative Social Activity	T = 2.094 NS	T = 10.87 *p* < 0.001	T = 9.274 *p* < 0.01
Excretion	T = 0.7731 NS	T = 10.98 *p* < 0.01	T = 3.286 NS
Foraging	T = 0.5482 NS	T = 2.340 NS	T = 0.1019 NS
Inactive	T = 0.0678 NS	T = 0.2288 NS	T = 0.3140 NS
Movement on Land	T = 0.2854 NS	T = 0.1771 NS	T = 0.0660 NS
Scenting	T = 0.4606 NS	T = 1.7107 NS	T = 0.2749 NS

### Appendix H.4. The Interquartile Range for Control Period

IQR values are presented to illustrate the magnitude and direction of variability, complementing the Fligner–Killeen test results shown in Table A10.

**Table A12:** The interquartile range is shown for the male, female and calf in control period. Cells marked with NA indicate behaviors observed less than 5%.

Behavior	Male	Female	Calf
Accelerated Movement on Land	0.3588	NA	3.661
Activity in Water	37.47	18.14	13.98
Affiliative Social Activity	NA	5.258	3.244
Foraging	48.33	29.28	9.403
Inactive	7.037	5.760	18.75
Movement on Land	7.932	9.819	3.944
Scenting	8.726	13.48	8.100

### Appendix H.5. The Interquartile Range for Test Period

IQR values are presented to illustrate the magnitude and direction of variability, complementing the Fligner–Killeen test results shown in Table A11.

**Table A13:** The interquartile range is shown for the male, female and calf in test period. Cells marked with NA indicate behaviors observed less than 5%.

Behavior	Male	Female	Calf
Accelerated Movement on Land	NA	0.4641	1.068
Activity in Water	14.47	11.43	27.39
Affiliative Social Activity	NA	0.1293	4.410
Excretion	1.229	1.055	NA
Foraging	37.10	29.28	22.03
Inactive	5.539	7.011	2.306
Movement on Land	3.061	3.368	4.830
Scenting	15.66	9.578	7.740
